# GSH-Activatable Aggregation-Induced Emission Cationic Lipid for Efficient Gene Delivery

**DOI:** 10.3390/molecules28041645

**Published:** 2023-02-08

**Authors:** Yue-Rui Yuan, Qiang Liu, Deyu Wang, Yu-Dan Deng, Ting-Ting Du, Wen-Jing Yi, Sheng-Tao Yang

**Affiliations:** 1 Key Laboratory of General Chemistry of the National Ethnic Affairs Commission, College of Chemistry and Environment, Southwest Minzu University, Chengdu 610041, China; 2 Key Laboratory of Pollution Control Chemistry and Environmental Functional Materials for Qinghai-Tibet Plateau of the National Ethnic Affairs Commission, School of Chemistry and Environment, Southwest Minzu University, Chengdu 610041, China

**Keywords:** gene delivery, cationic lipids, aggregation-induced emission, reduction responsive, quinoline-malononitrile

## Abstract

The key to gene therapy is the design of biocompatible and efficient delivery systems. In this work, a glutathione (GSH)-activated aggregation-induced-emission (AIE) cationic amphiphilic lipid, termed QM-SS-KK, was prepared for nonviral gene delivery. QM-SS-KK was composed of a hydrophilic biocompatible lysine tripeptide headgroup, a GSH-triggered disulfide linkage, and a hydrophobic AIE fluorophore QM-OH (QM: quinoline-malononitrile) tail. The peptide moiety could not only efficiently compact DNA but also well modulate the dispersion properties of QM-SS-KK, leading to the fluorescence-off state before GSH treatment. The cleavage of disulfide in QM-SS-KK by GSH generated AIE signals in situ with a tracking ability. The liposomes consisted of QM-SS-KK, and 1,2-dioleoylphosphatidylethanolamine (DOPE) (QM-SS-KK/DOPE) delivered plasmid DNAs (pDNAs) into cells with high efficiency. In particular, QM-SS-KK/DOPE had an enhanced transfection efficiency (TE) in the presence of 10% serum, which was two times higher than that of the commercial transfection agent PEI25K. These results highlighted the great potential of peptide and QM-based fluorescence AIE lipids for gene delivery applications.

## 1. Introduction

Gene therapy is a potent technique to treat diseases with a genetic origin through the intracellular use of exogenous genetic materials [1]. However, naked genetic materials can spontaneously hard cross the negatively charged cell membrane. Moreover, these genetic materials may suffer from rapid degradation by nucleases during the transport processes [2]. Both reasons lead to the poor internalization efficiency of genetic materials. Therefore, the design of safe and efficient gene vectors is indispensable for such therapeutic purposes [3]. Viral and nonviral ones are commonly used gene vehicles. Nonviral carriers have lower efficiencies compared with viral ones. However, nonviral vectors have many advantages, including the low toxicity, the excellent biocompatibility, the easy to manufacture at a large scale, etc. Cationic lipids make up an important category of nonviral gene vectors for gene transfection applications [4,5]. Typically, the cationic lipids are composed of three fragments: a hydrophilic head group, a hydrophobic tail, and a linker to connect the former two parts [6,7,8]. Each fragment can seriously affect the stability, disassembly, and phase behavior of the cationic lipids.

Vector-mediated gene transfection is a multistep process, and the vector needs to solve the dilemma of potent extracellular DNA protection versus intracellular easy release [9]. Moreover, understanding the gene transfection step and the transport mechanism is also extremely important for gene delivery. Multivalent cationic lipids using biocompatible amnio acids or peptides as polar domains have strong electrostatic interactions with negatively charged nucleic acids, which lead to better DNA condensation and more cellular uptakes [10,11,12,13,14]. To facilitate the intracellular DNA release, gene delivery vectors with biological reduction-responsive capacities have received increasing interest. There are significantly different GSH concentrations in the extracellular and intracellular environments [15,16]. Disulfide-bridged vectors can be disintegrated in a cytoplasm through the specific response to the redox potential of GSH, resulting in the detachment of DNAs from vectors through thiol-disulfide exchange reactions [12,13,14,15,16]. In addition, the introduction of reduction-responsive and biodegradable disulfide into the skeleton of the cationic lipids could also alleviate their cytotoxicity. To track the gene transfection process, fluorescent dyes, such as naphthalimide [2,17], tetraphenylethylene [18,19,20], and triphenylamine [21,22], have been used to functionalize the vectors. Among them, vectors with aggregation-induced-emission (AIE) characteristics have a strong emission capability in aggregation states, which can overcome the drawback of the aggregation-caused quenching (ACQ) effect, and have great potential in biomolecule detecting and delivery tracking [23,24,25]. As a new class of AIE building block, the chromophore quinoline-malononitrile (QM) has several prominent features, such as red to near-infrared (NIR) emission, marked photostability, excellent brightness, and good biocompatibility [26]. It holds great potential in organelle imaging, biomolecule detecting, and biological process monitoring [26,27,28]. Recently, Zhu et al. developed two excellent enzyme-activatable AIE fluorescent probes, QM-HSP-CPP and QM-GFTN, which are based on QM [29,30]. These probes were successfully applied to the fluorescent diagnosis of pancreatic cancer and autophagy, respectively. The peptide unit improved the dispersibility of probes and turned off the fluorescence. After the hydrophilic peptide was cleaved by a specific enzyme, the fluorescence recovered with a high signal-to-noise ratio [29,30].

**Scheme 1:**
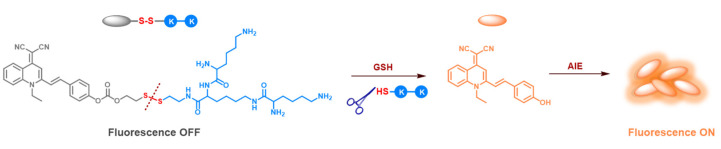
Illustration of GSH-activatable QM-SS-KK.

Inspired by these examples, we designed a GSH-activated AIE cationic lipid, termed QM-SS-KK, by using a lysine-based tripeptide as the polar group, a disulfide as the linking moiety, and a hydrophobic QM-OH core with AIE characteristics as a tail. The peptide moiety could not only efficiently bind DNA to facilitate the DNA condensation and cellular uptake but also well modulate the dispersion properties of QM-SS-KK, resulting in the initial fluorescence-off state. After the disulfide in QM-SS-KK was cleaved by GSH, it generated in situ AIE signals as the fluorescence-on state (Scheme 1). Its photophysical properties, DNA interactions, cytotoxicity, and gene transfection activity and the intracellular fate of the lipoplexes were investigated to evaluate the potential of QM-SS-KK for gene delivery applications.

## 2. Result and Discussion

### 2.1. Syntheses of Lipid QM-SS-KK

**Scheme 2:**
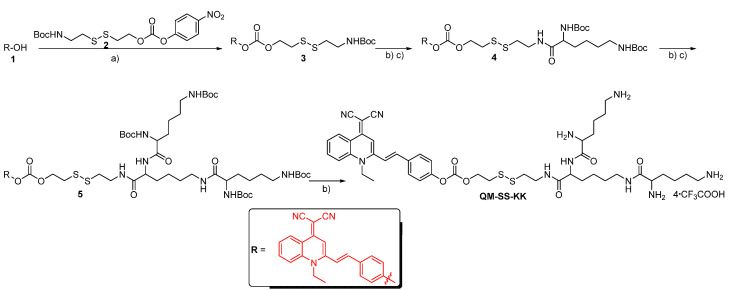
Synthesis of QM-SS-KK. Reagents and conditions: (**a**) DMAP, (**b**) CF_3_COOH, CH_2_Cl_2_, and (**c**) *N^α^*,*N^ε^*-di-Boc-l-lysine, isobutyl chloroformate, NMM.

QM-SS-KK was synthesized following the route in Scheme 2. Compounds **1** [31], and **2** [32] were prepared according to the literature protocols. Compound **3** was obtained by coupling compound **1** and **2** in the presence of 4-dimethylaminopyridine (DMAP). Coupling the deprotection product of compound **3** with *N^α^*,*N^ε^*-di-Boc-l-lysine by using isobutyl chloroformate and *N*-methyl morpholine (NMM) yielded compound 4. Subsequently, precursor **5** was obtained from **4** through the same procedures in the preparation from **3** to **4**. Finally, the Boc groups of **5** were removed to produce the target lipid QM-SS-KK by using trifluoroacetic acid in anhydrous CH_2_Cl_2_. The chemical structures of all new compounds were confirmed by NMR and high-resolution mass spectroscopy (HRMS).

### 2.2. Photophysical Characterization

**Figure 1:**
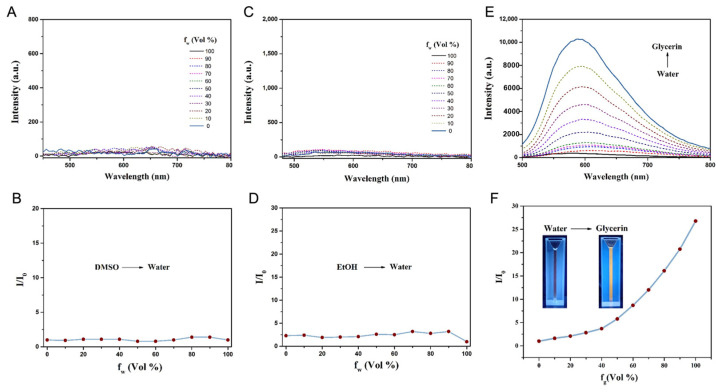
AIE spectra of QM-SS-KK in various solvents of different water fractions (*f*_w_). Emission spectra of QM-SS-KK (10 × 10^−6^ mol/L) in DMSO-water system (**A**), EtOH-water system (**C**), and glycerin-water system (**E**) (λ_ex_ = 455 nm). *I*/*I*_0_ plots of QM-SS-KK (**B**,**D**,**F**). *I* refers to the fluorescence intensity of fluorophore at 590 nm. *I*_0_ refers to the fluorescence intensity of fluorophore in 0% of water for (**A**) and (**C**), and 0% of glycerin for (**E**).

The photophysical properties of QM-SS-KK were investigated by photoluminescence spectrometry. It showed the maximum absorption peak at 430 nm in water and negligible fluorescence emission intensity (<100) at a concentration of 10^−6^ mol L^−1^ (Figure S1). The absorbance at 430 nm fitted a linear relationship with the concentration of QM-SS-KK in water (Figure S1). To further verify the initial fluorescence-off state of QM-SS-KK, the spectral properties of QM-SS-KK in a DMSO-water and ethanol (EtOH)-water mixture solution were detected. As shown in Figure 1A–D, QM-SS-KK showed a negligible fluorescence signal in both systems at all water fractions, indicating that the incorporation of hydrophilic lysine tripeptide could effectively eliminate the background signal. QM-SS-KK in the initial fluorescence-off state was ascribed to the free intramolecular motion (RIM) [29]. On the other hand, the restriction of RIM could block the nonirradiation and generate AIE signals [29,30,33]. Thus, the AIE phenomenon of QM-SS-KK was examined by restricting the molecular motion by enhancing the fraction of glycerin (*f*_g_). As presented in Figure 1E, the free motion of QM-SS-KK was restricted in the high-viscosity system, and the excited energy was released in the form of a radiative transition. In particular, the fluorescence intensity of QM-SS-KK at *f*_g_ = 99.9% was ≈25-fold of its initial intensity, which suggested that QM-SS-KK had a classical AIE characteristic (Figure 1F). In addition, the solution color changed from colorless to orange under UV lamp illumination at 365 nm (Figure 1F).

### 2.3. Agarose-Gel Retardant Assays of Liposomes and Lipoplexes

**Figure 2:**
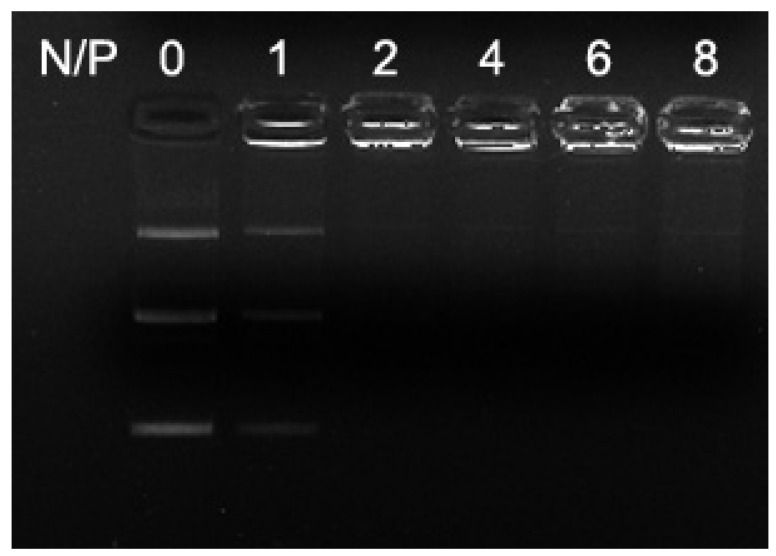
Electrophoretic gel retardation assays of pDNA binding for QM-SS-KK/DOPE/pDNA complexes under various N/P ratios.

The cationic liposome was prepared from lipid QM-SS-KK through the membrane hydration method in the presence of the helper lipid DOPE, and a lipid/DOPE ratio of 1:1 was adopted in our experiments. To evaluate the DNA-encapsulating capacity of QM-SS-KK/DOPE, the agarose-gel-retardant assay was employed (Figure 2). QM-SS-KK/DOPE exhibited good nucleic-acid-binding ability. The minimum N/P (the protonable amino groups of the lipid (N) to the phosphate groups (P) of DNA) ratio was 2. The strong complexation indicated that QM-SS-KK/DOPE could efficiently encapsulate pDNA and might be used as a pDNA delivery candidate for further evaluation.

### 2.4. Hydrodynamic Radii and Zeta Potentials of Lipoplexes

**Figure 3:**
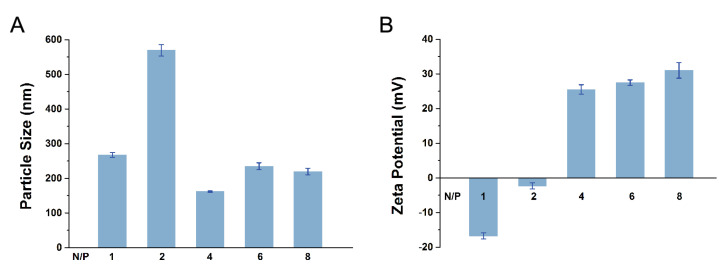
Changes in particle sizes (**A**) and zeta potentials (**B**) of the lipoplexes, along with the increases in N/P ratios. Data represent mean ± SD (*n* = 3).

To examine the hydrodynamic size and zeta potential value of the as-prepared lipoplexes, the dynamic light scattering (DLS) measurements of the lipoplexes were performed. As shown in Figure 3A, QM-SS-KK/DOPE could compact DNA into nanosized condensates with diameters in the range of 161 ± 2 to 569 ± 16 nm, which was intensely regulated by the N/P ratio. QM-SS-KK/DOPE could form lipoplexes nanoparticles with size of ~200 nm above the N/P ratio of 4. Subsequently, the surface potentials of the resulting nanosized condensates were examined by using DLS (Figure 3B). The zeta potentials increased from −17 ± 1 mV to 31 ± 2 mV, and the N/P ratio increased from 1 to 8. The zeta potential data demonstrated that the charge reversal from negative to positive was initiated at the N/P ratio of 4 (Figure 3B), indicating that DNA was fully condensed at the N/P values of 2–4.

### 2.5. Reduction-Triggered Cleavage of Lipid and Liposome

**Figure 4:**
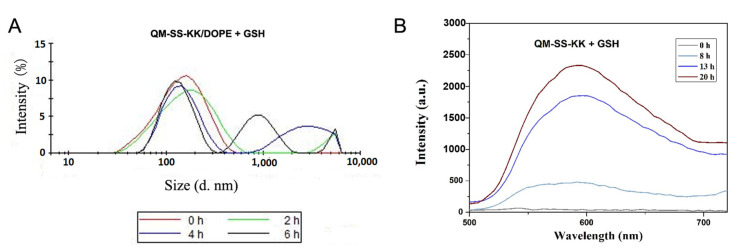
DLS assay of QM-SS-KK/DOPE (**A**) and fluorescence assay of QM-SS-KK (**B**) in the presence of 10 mM of GSH after different incubation periods.

To investigate the GSH reduction-sensitive capacity of QM-SS-KK, DLS measurements were first performed to monitor the size changes in the QM-SS-KK/DOPE by incubating it with 10 mM of GSH for different lengths of time (0–6 h). The particle size of QM-SS-KK/DOPE gradually increased with an increasing incubation time (Figure 4A), indicating that GSH would reorganize the structure of the liposome and facilitate the DNA release. Moreover, fluorescence spectrometry was used to monitor the time-dependent degradation of QM-SS-KK in the presence of 10 mM of GSH. With the elongation of incubation time (0–20 h), the fluorescence emission of QM-SS-KK at 590 nm gradually increased (Figure 4B). This showed that GSH could cleave the disulfide in QM-SS-KK and light up the AIE fluorescence. The results were consistent with the DLS measurements.

### 2.6. Cytotoxicity Assay

**Figure 5:**
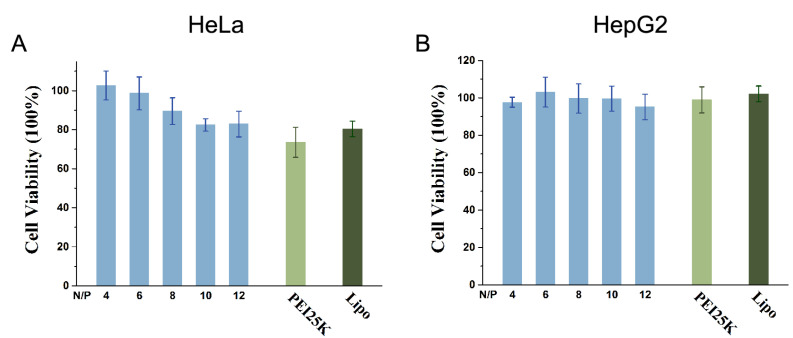
Cytotoxicity of the QM-SS-KK/DOPE/pDNA complexes prepared at various N/P ratios in a serum-free medium of HeLa cells (**A**) and HepG2 cells (**B**). PEI25K and lipofectamine 2000 were used as controls. Data represent mean ± SD (*n* = 3).

To assess the biocompatibility of the prepared lipoplexes, the cytotoxicity was measured by using MTT (3-(4, 5-dimethylthiazolyl-2)-2, 5-diphenyltetrazolium bromide) assay, using HeLa and HepG2 cell lines as the model organisms in a serum-free medium. After 24 h of incubation, the cytotoxicity was examined through cell viability, where lipofectamine 2000 and PEI25K were used as controls. According to the results in Figure 5, cells treated with QM-SS-KK/DOPE/pDNA complexes showed >80% viability in both cell lines even at the high N/P ratio of 12, indicating that the novel lipoplexes were of low cytotoxicity, and therefore, its biocompatibility was good enough for further transfection.

### 2.7. In Vitro Transfection

**Figure 6:**
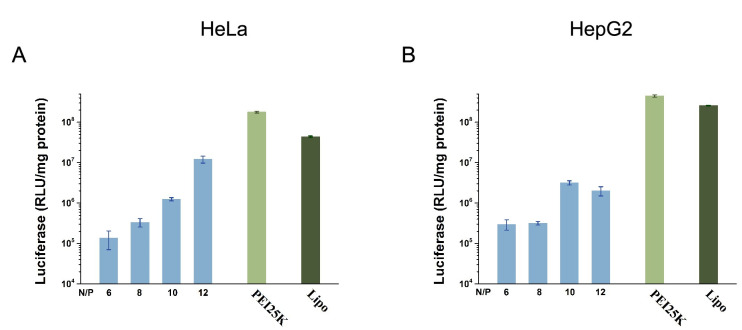
Luciferase expression induced by QM-SS-KK/DOPE-pGL3 plasmid complex of different N/P ratios in HeLa cells (**A**) and HepG2 cells (**B**) in a serum-free medium. PEI25K (N/P = 10) and lipofectamine 2000 were taken as controls. Data represent mean ± SD (*n* = 3).

The gene transfection efficiency (TE) of the QM-SS-KK in vitro was studied with HeLa and HepG2 cells. The pGL3 plasmid was adopted as the luciferase reporter gene, while lipofectamine 2000 and PEI25K were taken as controls. Cells were transfected with QM-SS-KK/DOPE-pGL3 plasmid complexes of different N/P ratios for 24 h in a serum-free medium. A subsequent analysis through quantitative luciferase expression showed that the TE of the QM-SS-KK/DOPE is dramatically increased by increasing the N/P ratio in HeLa cells (Figure 6A). In addition, QM-SS-KK/DOPE also showed better TE at higher N/P ratio in HepG2 cells. However, the TE values of QM-SS-KK/DOPE were lower than those of lipofectamine 2000 and PEI25K (Figure 6A,B).

**Figure 7:**
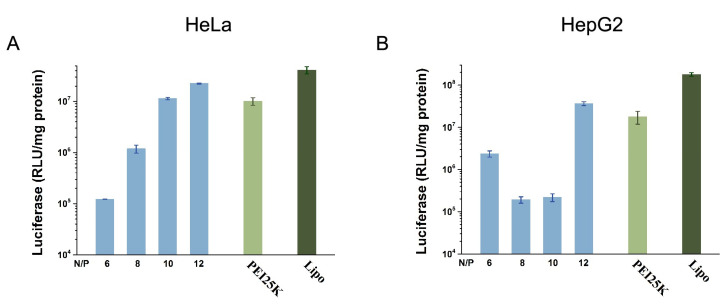
Luciferase expression induced by QM-SS-KK/DOPE of various N/P ratios in HeLa cells (**A**) and HepG2 cells (**B**) with the supplement of 10% FBS. PEI25K (N/P = 10) and lipofectamine 2000 were taken as controls. Data represent mean ± SD (*n* = 3).

To assess the impacts of the serum on the transfection efficiency of a liposome, the transfection with QM-SS-KK/DOPE in the same cell lines was further studied by adding 10% fetal bovine serum (FBS) (Figure 7). Again, PEI25K and lipofectamine 2000 were adopted as controls. In HeLa cells, the TEs of QM-SS-KK/DOPE showed similar trends as those in a serum-free medium, where the TE values increased as the N/P ratio increased. In addition, QM-SS-KK/DOPE showed better transfection performance in a serum medium (Figure 7A). Furthermore, the TE of QM-SS-KK/DOPE was also investigated in HepG2 cells (Figure 7B). The optimum TE of QM-SS-KK/DOPE (N/P = 12) was dramatically increased, specifically 11.5-fold, compared with the TE of QM-SS-KK/DOPE (N/P = 10) without serum (Figure 6B and Figure 7B). In contrast, the TE of PEI25K and lipofectamine 2000 both decreased in two cell lines in a serum medium, but PEI25K showed a sharper way (Figure 7A,B). Impressively, in both cell lines of QM-SS-KK/DOPE showed two times higher TE than PEI25K with a serum circumstance. The results showed the good serum tolerance of QM-SS-KK.

### 2.8. CLSM (Confocal Laser Scanning Microscopy) Measurements

**Figure 8:**
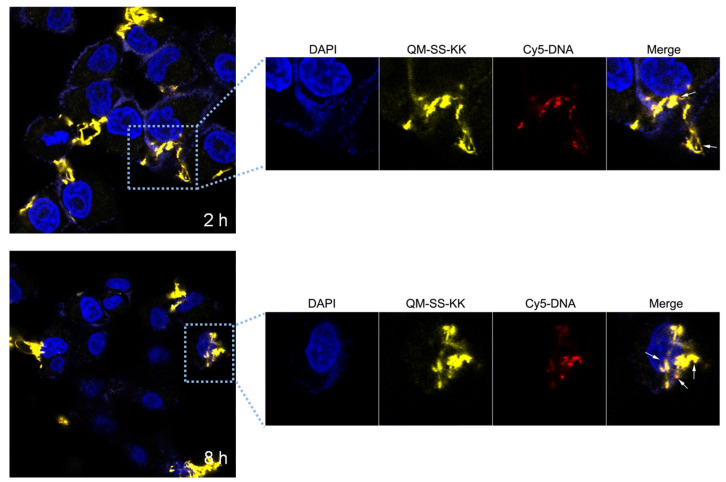
CLSM images of HeLa cells after the transfection with Cy5-labeled DNA by QM-SS-KK/DOPE at N/P ratio of 6 for different lengths of time (red: Cy5-labeled pDNA; yellow: QM-SS-KK/DOPE; blue: DAPI-stained cell nuclei).

AIE has been regarded as a powerful tool for tracking the gene transport process. In this study, Cy5-labeled dsDNA, which emits red fluorescence (red) was wrapped by QM-SS-KK/DOPE (yellow) to form lipoplexes (N/P = 6) and was incubated with HeLa cells. In addition, the cell nuclei of the cells were stained with DAPI (4,6-diamidino-2-phenylindole), which emits blue fluorescence (blue). CLSM was adopted to visualize the intracellular fate of the lipoplexes. Colocalization CLSM images were taken at 2 h and 8 h. As shown in Figure S2, after incubation for 2 h with the QM-SS-KK/DOPE/Cy5-pDNA lipoplexes, a portion of the cells was stained by QM-SS-KK/DOPE (yellow) and Cy5-labeled dsDNA (red), suggesting the successful cellular uptake of the lipoplexes. With the extension of transfection time, more yellow and red colors had separated, resulting in an increase in the delivered DNA around the cell nuclei (red fluorescence represents the labeled pDNA). The results indicated the dissociation of the vector and the DNA (Figure 8). Nevertheless, most DNAs were still binding with the liposomes at 8 h. The poor DNA release should be attributed to its strong DNA complex affinity. Hence, it is vital to balance the DNA-binding ability and TE while designing peptide-modified gene vectors.

## 3. Experimental Section

### 3.1. Materials

*N^α^*,*N^β^*-di-Boc-l-lysine was obtained from GL Biochem (Shanghai, China). 1,2-Dioleoyl-*sn*-glycero-3-phosphoethanolamine (DOPE) was bought from Avanti Polar Lipids (Alabaster, AL, USA). Lipofectamine 2000 was obtained from Invitrogen Life Technologies (Waltham, MA, USA). Compounds **1** and **2** were prepared following the literature procedures [31,32]. Other chemicals and instruments were the same as those in the literature [13,14,17].

### 3.2. Synthesis of Lipid QM-SS-KK

#### 3.2.1. Preparation of Compound **3**

Compound **1** (679 mg, 2 mmol) and DMAP (48.9 mg, 0.4 mmol) were dissolved in DMF (10 mL), and thereafter, compound **2** (920 mg, 2.2 mmol) in 20 mL of DMF was added dropwise to the solution at 0 °C. Upon stirring at 0 °C for 1 h, the mixture was further stirred overnight at room temperature. The solvent was evaporated under reduced pressure, and the crude product was purified by silica chromatography with PE/EtOAc (1:1) to produce compound **3** (orange solid, 383 mg, yield of 31%). ^1^H NMR (400 MHz, CDCl_3_) δ 9.01 (d, *J* = 8.4 Hz, 1H), 7.77 (t, *J* = 7.6 Hz, 1H), 7.65 (d, *J* = 8.8 Hz, 1H), 7.58 (d, *J* = 8.2 Hz, 2H), 7.40 (t, *J* = 7.6 Hz, 1H), 7.26 (d, *J* = 3.8 Hz, 2H), 7.05 (d, *J* = 8.8 Hz, 2H), 4.97 (s, 1H), 4.53 (t, *J* = 6.4 Hz, 2H), 4.41 (q, *J* = 6.8 Hz, 2H), 3.47 (d, *J* = 5.0 Hz, 2H), 3.04 (t, *J* = 6.4 Hz, 2H), 2.85 (t, *J* = 6.0 Hz, 2H), 1.57 (t, *J* = 6.8 Hz, 3H), 1.45 (s, 9H). ^13^C NMR (101 MHz, CDCl_3_) δ 155.72, 153.31, 153.02, 152.05, 147.62, 138.86, 138.00, 133.36, 132.84, 128.82, 126.70, 124.66, 121.70, 121.40, 120.14, 120.02, 119.04, 116.23, 107.75, 79.56, 66.53, 60.37, 51.16, 44.12, 39.25, 38.55, 36.57, 28.37, 21.04, 14.19, 13.94. HRMS (ESI): [M + H]^+^ calcd for C_32_H_35_N_4_O_5_S_2_: 619.2043; found: 619.2045.

#### 3.2.2. Preparation of Compound **4**

Compound **3** (185.5 mg, 0.3 mmol) was dissolved in 3 mL of anhydrous dichloromethane. Thereafter, 3 mL of CF_3_COOH was added dropwise to the solution at 0 °C. Upon 6 h of stirring, the solvent was evaporated under reduced pressure to yield the deprotection product. Subsequently, *N^α^*,*N*^ε^-di-Boc-l-lysine (346 mg, 1 mmol) was dissolved in 30 mL of CH_2_Cl_2_, followed by the gradual addition of *N*-methylmorpholine (1 mmol) and isobutyl chloroformate (16.4 mg, 0.12 mmol). After 1 h of stirring at 0 °C, the deprotection product of **3** that had been dissolved in CH_2_Cl_2_ (10 mL) was added dropwise into the reactant and stirred overnight. The mixture was washed twice with 20 mL of saturated aqueous NaHCO_3_ solution and once with 20 mL of brine. The organic phase was dried over Na_2_SO_4_, filtered, and concentrated to give the orange solid. After the column chromatography purification with silica gel, the orange solid was obtained as compound **4** (142 mg, yield of 55.8%). ^1^H NMR (400 MHz, CDCl_3_) δ 9.10 (d, *J* = 8.4 Hz, 1H), 7.77 (t, *J* = 7.8 Hz, 1H), 7.63 (d, *J* = 8.8 Hz, 1H), 7.60 (d, *J* = 8.2 Hz, 2H), 7.45 (t, *J* = 7.6 Hz, 1H), 7.31 (d, *J* = 10.6 Hz, 2H), 7.11–7.02 (m, 2H), 5.16 (s, 1H), 4.54 (t, *J* = 6.6 Hz, 2H), 4.40 (q, *J* = 6.8 Hz, 2H), 4.04 (t, *J* = 10.2 Hz, 1H), 3.61 (t, *J* = 6.6 Hz, 2H), 3.10 (s, 2H), 3.04 (t, *J* = 6.6 Hz, 2H), 2.86 (t, *J* = 6.2 Hz, 2H), 1.88–1.79 (m, 2H), 1.59 (dt, *J* = 14.2, 7.4 Hz, 5H), 1.44 (s, 18H), 1.26 (m, 2H). ^13^C NMR (101 MHz, CDCl_3_) δ 172.41, 156.19, 153.41, 153.06, 152.04, 147.60, 138.86, 138.01, 133.35, 132.87, 128.83, 126.80, 124.70, 121.71, 121.44, 120.11, 120.04, 119.04, 116.17, 107.80, 80.07, 79.12, 66.56, 54.41, 51.32, 44.07, 39.86, 38.08, 37.81, 36.48, 31.90, 29.65, 28.44, 28.35, 22.58, 13.93. HRMS (ESI): [M + Na]^+^ calcd for C_43_H_54_N_6_NaO_8_S_2_: 869.3337; found: 869.3339.

#### 3.2.3. Preparation of Compound **5**

Compound **5** was prepared by following the same protocols as those for the preparation of compound **4.** The crude product was purified by silica chromatography with CH_2_Cl_2_/methanol (1:1) to produce the orange solid compound **5** (115.2 mg, yield of 58.9%). ^1^H NMR (400 MHz, CDCl_3_) δ 9.06 (d, *J* = 8.4 Hz, 1H), 7.76 (t, *J* = 7.8 Hz,1H), 7.62 (d, *J* = 8.8 Hz, 1H), 7.57 (d, *J* = 8.4 Hz, 2H), 7.45 (t, *J* = 7.6 Hz,1H), 7.29 (d, *J* = 10.6 Hz,2H), 7.04 (m, 2H), 5.57 (s, 1H), 4.98–4.72 (m, 3H), 4.51 (t, *J* = 6.4 Hz, 2H), 4.38 (q, *J* = 6.8 Hz, 2H), 4.22 (t, *J* = 6.4 Hz, 2H), 3.66–3.50 (m, 2H), 3.13–2.97 (m, 6H), 2.84 (t, *J* = 6.2 Hz, 2H), 1.86–1.63 (m, 6H), 1.62–1.46 (m, 9H), 1.42 (s, 36H), 1.28 (m, 6H). ^13^C NMR (101 MHz, CDCl_3_) δ 156.33, 153.46, 153.04, 152.07, 147.58, 138.89, 138.02, 133.34, 132.82, 128.80, 126.88, 124.71, 121.73, 121.47, 119.99, 119.02, 116.11, 112.49, 107.82, 79.00, 66.58, 44.03, 37.82, 37.69, 36.49, 29.66, 29.62, 29.53, 28.44, 28.39, 22.81, 22.67, 22.64, 13.94. HRMS (ESI): [M + Na]^+^ calcd for C_65_H_94_N_10_NaO_14_S_2_: 1325.6285; found: 1325.6286.

#### 3.2.4. Preparation of Lipid QM-SS-KK

Compound **5** (100 mg, 0.08 mmol) was dissolved in anhydrous CH_2_Cl_2_ (3 mL), followed by the addition of CF_3_COOH (3 mL) at 0 °C. Upon 6 h of stirring, the solvent was removed to produce the red product, which was washed twice by ether to obtain the title lipid QM-SS-KK as red solid (97.8 mg, yield of 90%). ^1^H NMR (400 MHz, CD_3_OD) δ 9.01 (d, *J* = 8.6 Hz, 1H), 7.99 (d, *J* = 9.0 Hz, 1H), 7.91–7.86 (t, *J* = 8.4 Hz 1H), 7.78 (d, *J* = 8.6 Hz, 2H), 7.53 (t, *J* = 8.2 Hz 1H), 7.42 (s, 2H), 7.29 (d, *J* = 8.4 Hz, 2H), 7.11 (s, 1H), 4.54 (dt, *J* = 12.8, 6.6 Hz, 4H), 4.32 (t, *J* = 8.4 Hz 1H), 3.96–3.81 (m, 2H), 3.63–3.47 (m, 2H), 3.26–3.14 (m, 2H), 3.07 (t, *J* = 6.4 Hz, 2H), 3.01–2.81 (m, 6H), 1.85 (m, 6H), 1.71–1.50 (m, 9H), 1.49–1.25 (m, 6H). ^13^C NMR (101 MHz, CD_3_OD) δ 176.57, 172.23, 157.72, 157.45, 157.20, 156.06, 152.97, 142.56, 141.91, 137.17, 137.04, 132.78, 128.33, 125.24, 125.09, 124.30, 121.11, 111.09, 70.28, 61.47, 57.55, 56.78, 56.36, 47.63, 42.94, 42.64, 41.01, 40.14, 35.23, 34.57, 34.48, 32.30, 30.55, 30.34, 26.63, 25.44, 24.77, 16.43. HRMS (ESI): [M + Na]^+^ calcd for C_45_H_62_N_10_NaO_6_S_2_: 925.4187; found: 925.4189.

### 3.3. Preparation of the Cationic Liposomes

Cationic liposomes were prepared from the cationic lipid and the helper lipid. Briefly, cationic lipid (0.0025 mmol) and neutral colipid (DOPE, 0.0025 mmol) were dissolved in a mixture solvent (chloroform and MeOH, 2.5 mL, *v*/*v* = 4:1) in autoclaved vials. The solvent was evaporated by a thin flow of moisture-free N_2_, and thereafter, the lipid film was dried under high vacuum for 8 h. After that, 2.5 mL of sterile deionized water was added and vortexed for 2 min. After removing the adhering lipid film, the mixture was sonicated for 10 min at room temperature to obtain the multilamellar vesicles (MLVs). MLVs were further sonicated by a probe sonifier in an ice bath to produce the corresponding cationic liposomes (1.0 mmol L^−1^) [13,14,17].

### 3.4. Agarose-Gel Retardation Assays

QM-SS-KK/DOPE/pDNA complexes of N/P ratios from 1 to 8 were prepared by adding the designed volumes of liposome to 0.125 μg of pUC-19 DNA. The theoretical N/P ratio represents the charge ratio of cationic lipid to nucleotide base (mole ratios) and was calculated by considering the average nucleotide mass of 330. After the incubation at 37 °C for 30 min, the complexes were electrophoresed. The 1.0% (*w*/*v*) agarose-gel containing GelRed and Tris-acetate (TAE) running buffer was adopted. The experiment was performed at 110 V for 30 min. DNA was imaged under UV irradiation at 312 nm in a GeLDoc System (BioRad Universal Hood II, BioRad, Hercules, CA, USA) [13,14,17].

### 3.5. Fluorescence Assays

The changes in the liposome fluorescence induced by GSH were monitored with a fluorescence spectrometer (FluoroMax-4, HORIBA, Piscataway, NJ, USA). The aqueous GSH solution (10 mM, 1 mL) was added with a 1 mM lipid solution (10 μL). After incubation at 37 °C for 8 h, 13 h, and 20 h, the fluorescence spectra were recorded. The excitation wavelength was set as 455 nm, and the emission band was centered at 590 nm.

### 3.6. Lipoplex Particle Sizes and Zeta Potentials

The hydrodynamic radii and the zeta potentials of the QM-SS-KK/DOPE/pDNA lipoplexes of different N/P ratios were recorded at 25 °C on a DLS system (Zetasizer Nano ZS, Malvern Instruments Led, Malvern, United Kingdom). The lipoplex dispersion was prepared by mixing QM-SS-KK/DOPE and pDNA (1 μg/mL) at designed N/P ratios in 1 mL of deionized water [13,14,17]. For the size of the reduction-triggered cleavage of liposome, liposome solution (1 mM, 25 μL) was incubated with 20 mM of GSH (25 μL) for different lengths of time (0–6 h), and thereafter, the particle size was detected by DLS assay.

### 3.7. MTT Cytotoxicity Assays

The cell viability changes of HeLa and HepG2 cells after the exposure to the liposomes and lipoplexes were measured by the MTT assay. About 7000 cells were inoculated into each well of 96-well plates. For lipoplexes, after a 24 h incubation, the optimized formulations of lipid/DOPE were complexed with 0.2 μg of DNA at different N/P ratios. The lipoplexes using lipofectamine 2000 and the polyplexes using PEI25K were taken as the controls [13,14,17].

### 3.8. In Vitro Transfection Assays

HeLa and HepG2 cells were inoculated into 24-well plates at 8 × 10^4^ cells per well in a complete medium (0.5 mL per well). A day prior to transfection experiments, the medium was discharged, and another 1 mL of fresh medium was added with/without the supplement of FBS. QM-SS-KK/DOPE/DNA complexes, containing 1 μg of plasmid DNA at designed N/P ratios, were introduced to each well for another 4 h incubation. The medium was again replaced by 0.5 mL of fresh medium, followed by another 24 h incubation at 37 °C. For luciferase assays, the cells were transfected by pGL3 plasmid DNA lipoplexes. The Picagene luciferase assay kit (Tokyo Ink, Tokyo, Japan) was adopted. The transfected cells were washed with PBS three times and lysed in a cell lysis buffer. The lysate was centrifuged at 4 °C and 4000 rpm for 2 min to collect the supernatant. The relative light unit (RLU) of chemiluminescence was determined with a luminometer (Turner Designs, 20/20, Promega, Madison, WI, USA), and the luminescent RLU values were normalized by the protein content, which was measured by the BCA (bicinchoninic acid) assay. Transfection with lipofectamine 2000/DNA complexes and PEI25K (N/P = 10, 1 μg DNA, diluted with the optimum medium) were taken as the positive controls [13,14,17].

### 3.9. Confocal Laser Scanning Microscopy (CLSM) Analysis

HeLa cells were inoculated in a 35 mm confocal dish at 2.5 × 10^5^ cells per well. After 24 h incubation, the medium was discharged, and a fresh complete culture medium was added. The complexes of QM-SS-KK/DOPE and Cy5-labeled pDNA (1 μg DNA per well) were added to the dish at a designed concentration. After the incubation for 2 h and 8 h, the cells were washed with PBS buffer three times and stained with DAPI for 15 min. The cells were subsequently rinsed with PBS buffer another three times. The CLSM imaging was recorded on a Zeiss, LSM 780 (Zeiss, Tokyo, Japan), equipped with a 40× objective lens. The excitation wavelength was set as 488 nm for QM-SS-KK (yellow), 650 nm for Cy5 (red), and 405 nm for DAPI (blue) [13,14,17].

## 4. Conclusions

In summary, an amphiphilic AIE-active lipid QM-SS-KK was prepared for gene delivery applications, which consisted of a hydrophilic biocompatible lysine tripeptide as the polar headgroup and the amphiphilic regulator, a GSH-cleavable disulfide as the reduction-active trigger, and a QM-OH scaffold as the hydrophobic tail and the AIE-fluorescence reporter. QM-SS-KK had good dispersity in an aqueous biosystem, where the off-fluorescence and high affinity for GSH were shown. The liposome QM-SS-KK/DOPE condensed pDNA into stable nanoparticles and delivered it into cells. These lipoplexes showed good serum resistance in that the TEs increased in the serum-containing medium, and the TEs of QM-SS-KK were twofold higher than that of the commercial transaction agent PEI25K. QM-SS-KK/DOPE visualized the real-time intracellular gene delivery and displayed the subsequent gene fate. The results demonstrated the effectiveness of QM-SS-KK with good biocompatible, serum-tolerant, and AIE-fluorescence characteristics. In future, the structural modification of cationic lipids should be further investigated to balance their DNA-binding ability and improve their transfection efficiency.

## Data Availability

Not applicable.

## Supplementary Materials

The following supporting information can be downloaded at https://www.mdpi.com/article/10.3390/molecules28041645/s1, Figure S1: (A) UV/vis absorption spectra of QM-SS-KK at various concentrations in deionized water. (B) The molar absorption coefficient of QM-SS-KK and its emission spectrum in deionized water; Figure S2. CLSM images of HeLa cells transfected with Cy5-labeled DNA by QM-SS-KK/DOPE at N/P ratio of 6 for different lengths of time (red: Cy5-labeled pDNA; yellow: QM-SS-KK/DOPE; blue: DAPI-stained cell nuclei).

## Sample Availability

Samples of the compounds are not available from the authors.
