# Supplementary material for: GSH-Activatable Aggregation-Induced Emission Cationic Lipid for Efficient Gene Delivery
Source: Molecules. 2023 Feb 8;28(4):1645. doi: 10.3390/molecules28041645 (PMC9963561; doi:10.3390/molecules28041645)
Supplement: Supplementary file 1 [file molecules-28-01645-s001.zip › molecules-2182660-supplementary.pdf]

Supporting Information

Article

GSH-Activatable Aggregation-Induced Emission Cationic Lipid for Efficient Gene Delivery

Yue-Rui Yuan [a], Qiang Liu [a], Deyu Wang [a], Yu-Dan Deng [a], Ting-Ting Du [a], Wen-Jing Yi [b]* and Sheng-Tao Yang [b]*

[a] Key Laboratory of General Chemistry of the National Ethnic Affairs Commission, College of Chemistry and Environment, Southwest Minzu University, Chengdu 610041, P. R. of China; [b] Key Laboratory of Pollution Control Chemistry and Environmental Functional Materials for Qinghai-Tibet Plateau of the National Ethnic Affairs Commission, School of Chemistry and Environment, Southwest Minzu University, Chengdu 610041, China

* Correspondence: ywjchem@163.com (W.-J.Y.); yangst@pku.edu.cn (S.-T.Y.); tel.: +86-8552-2315 (W.-J.Y.); tel: +86-85570-9707 (S.-T.Y.)

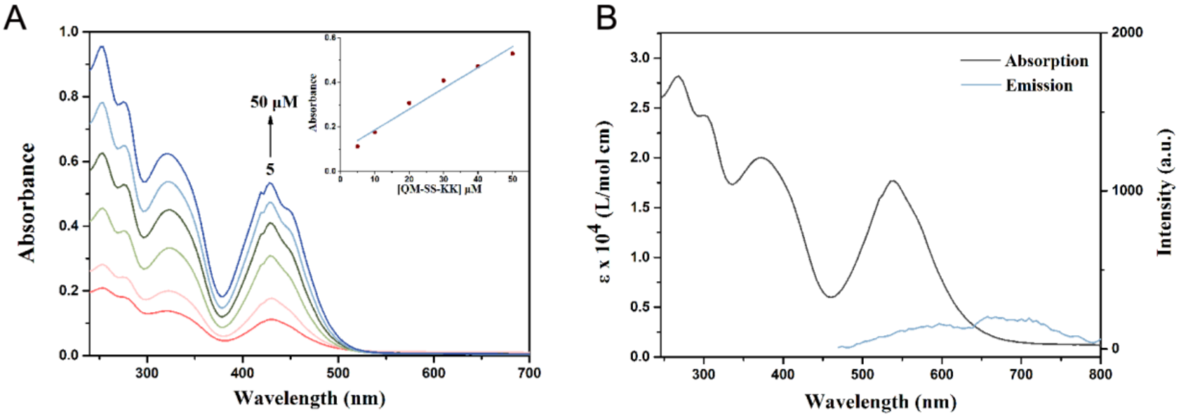

Figure S1. Spectroscopic characterizations of QM-SS-KK. (A) UV/vis absorption spectra of QM-SS-KK at various concentrations in deionized water. Inset: The plot of concentration of QM-SS-KK vs. absorbance at 430 nm; (B) The molar absorption coefficient of QM-SS-KK and its emission spectrum in deionized water.

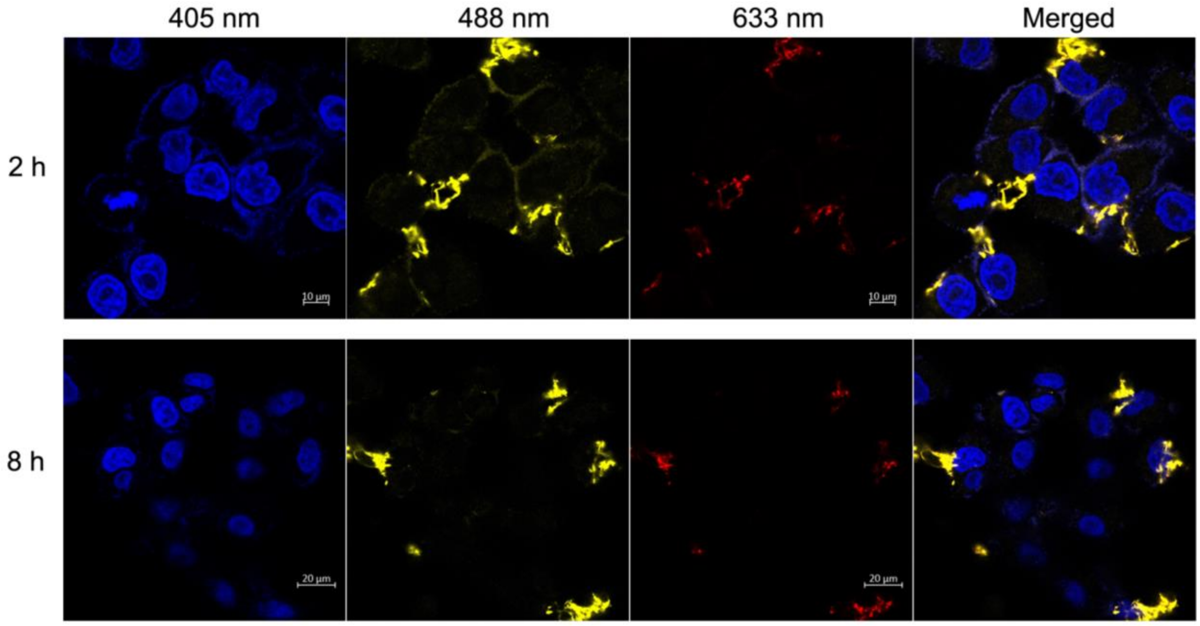

Figure S2. CLSM images of HeLa cells transfected with Cy5-labeled DNA by QM-SS-KK/DOPE at N/P ratio of 6 for different time. (Red: Cy5-labeled pDNA; yellow: QM-SS-KK/DOPE; blue: DAPI-stained cell nuclei).
